# Setmelanotide in Bardet‐Biedl Syndrome: A 52‐Week Comparison of Phase 3 Trial Participants With a Matched Registry Cohort

**DOI:** 10.1002/oby.70125

**Published:** 2026-02-17

**Authors:** Jesús Argente, Andrea M. Haqq, Jan Luca Schorfheide, Nicolas Touchot, Caroline Huber, Urs Wiedemann, Jeremy Pomeroy, the Phase 3 BBS Trial Investigators, Wendy Chung, Karine Clément, Hélène Dollfus, Elizabeth Forsythe, Robert M. Haws, Gabriel Á. Martos‐Moreno, Christine Poitou, Jack A. Yanovski

**Affiliations:** ^1^ Department of Pediatrics and Pediatric Endocrinology University Hospital Niño Jesús, Research Institute La Princesa Madrid Spain; ^2^ Department of Pediatrics Universidad Autónoma de Madrid Madrid Spain; ^3^ Centro de Investigación Biomédica en Red de Fisiopatología de la Obesidad y Nutrición (CIBEROBN), Instituto de Salud Carlos III Madrid Spain; ^4^ IMDEA, Food Institute, Campus of International Excellence Universidad Autónoma de Madrid (CEIUAM) + Spanish National Research Council (CSIC) Madrid Spain; ^5^ Division of Pediatric Endocrinology University of Alberta Edmonton Alberta Canada; ^6^ stradoo Gmbh Munich Germany; ^7^ Rhythm Pharmaceuticals, Inc. Boston Massachusetts USA; ^8^ Marshfield Clinic Research Institute Marshfield Wisconsin USA

**Keywords:** Bardet‐Biedl syndrome, indirect treatment comparisons, setmelanotide, weight outcomes

## Abstract

**Objective:**

This analysis aimed to assess the efficacy of setmelanotide over 52 weeks in patients with Bardet‐Biedl syndrome (BBS) compared with an external natural history cohort from the international Clinical Registry Investigating BBS (CRIBBS).

**Methods:**

Patients with BBS ≥ 6 years of age (*n* = 29) treated with setmelanotide for 52 weeks in a phase 3 trial (NCT03746522) and a propensity‐score matched external control cohort (*n* = 58) from CRIBBS were included. Responder rate at week 52 was defined as ≥ 0.3‐point decrease in BMI *z*‐score (patients ≤ 18 years) or ≥ 10% body weight reduction (adults). Secondary outcomes included changes in BMI, BMI *z*‐score, and body weight.

**Results:**

A significantly greater proportion of patients treated with setmelanotide met the primary endpoint compared with control patients (58.6% vs. 6.9%; *p* < 0.001). In pediatric patients, 71.4% achieved the primary endpoint vs. 10.7% of controls (*p* < 0.001); in adults, corresponding numbers were 46.7% vs. 3.3% (*p* = 0.001). Secondary outcomes demonstrated consistent benefits of setmelanotide treatment. Sensitivity analysis using inverse probability of treatment weighting confirmed these findings.

**Conclusions:**

This indirect comparison provides additional strong evidence that setmelanotide significantly improves weight outcomes in patients with BBS. These findings further support its clinical benefit over 52 weeks in managing obesity associated with BBS.

**Trial Registration:** ClinicalTrials.gov identifier: NCT03746522

## Study Importance

**What is already known?:**

- Bardet‐Biedl syndrome (BBS) is characterized by dysfunction in the hypothalamic melanocortin‐4 receptor (MC4R) pathway, potentially leading to hyperphagia and early‐onset obesity. This substantially impacts quality of life and increases the risk of obesity‐related complications.
- Setmelanotide, an MC4R agonist that is approved in Europe, Canada, and the United States, has shown its efficacy in hunger and weight reductions in patients with BBS and obesity in a phase 3 clinical trial with a 14‐week double‐blind, placebo‐controlled period followed by a 52‐week open‐label period.

**What does this study add?:**

- In absence of a placebo‐controlled comparison beyond 14 weeks in the phase 3 clinical trial, an indirect treatment comparison over 52 weeks was performed using matched controls from the international Clinical Registry Investigating BBS (CRIBBS).
- The indirect treatment comparison confirmed statistically and clinically meaningful reductions in BMI and body weight in pediatric and adult patients with BBS over 52 weeks compared with usual care, with consistent results across sensitivity analyses, reinforcing the robustness of the findings.

**How might these results change the direction of research or focus of clinical practice?:**

- These findings support the therapeutic value of setmelanotide and its use in managing obesity associated with BBS, potentially leading to improved quality of life and mitigating long‐term obesity‐related complications.

## 1 Introduction

Bardet‐Biedl syndrome (BBS) is a rare autosomal recessive disease classified among the ciliopathies [1]. Its estimated prevalence ranges from 1 in 100,000 to 1 in 160,000 in North America and Europe [1, 2], but it can be substantially higher, with 1 in 13,500 to 1 in 18,000 in genetically isolated populations, such as the nomadic Bedouins and remote Newfoundland communities [3, 4].

To date, causative variants in at least 26 genes and four modifiers have been identified in the pathogenesis of BBS [5]. These genes are involved in ciliary function, accounting for the syndrome's broad clinical spectrum due to their roles in multiple biological pathways [5]. A key feature of BBS is dysfunction of the hypothalamic melanocortin‐4 receptor (MC4R) pathway, which regulates appetite and energy balance [6]. Impaired signaling in this pathway may lead to hyperphagia, an insatiable hunger due to reduced satiety, which contributes to abnormal eating and persistent food‐seeking behaviors, and may result in early‐onset obesity [7, 8, 9, 10].

Hyperphagia is a severe manifestation and hallmark of BBS [11, 12, 13]. Its severity correlates strongly with obesity, which together are perceived as two of the most distressing aspects of BBS by parents of affected children [9, 12]. Both hyperphagia and obesity typically emerge early in the disease course, which may support earlier diagnosis and management of BBS [12]. Hyperphagia is typically unresponsive to diet and lifestyle modifications due to its neurological and behavioral underpinnings [7, 8, 9, 10]. Rapid weight gain is observed early in childhood with rates of overweight or obesity exceeding 90% after 5 years of age. Many patients display persistent overweight/obesity through adolescence [9].

Additional symptoms of BBS may include developmental delay, intellectual disability (in a minority of patients), and multiple organ involvement, such as retinal dystrophy, renal dysfunction, hypogonadism, and cardiac abnormalities [8, 14, 15, 16].

Early‐onset obesity linked to genetic causes such as BBS is associated with an increased risk of severe complications in childhood and adulthood, including sleep apnea, early insulin resistance, prehypertension/hypertension, metabolic‐associated steatotic liver disease (MASLD), and dyslipidemia [17, 18]. Moreover, infants and children with early obesity frequently face considerable psychosocial challenges, including impaired social development, early‐onset body image concerns, and weight bias and stigma. In consequence, caregiver burden is often substantial [12, 17, 18, 19].

Setmelanotide is a selective MC4R agonist that is designed to restore MC4R pathway function in BBS, thereby reducing hunger and promoting weight loss through decreased caloric intake and increased energy expenditure [20]. In Europe, setmelanotide is indicated for the control of hunger and the treatment of obesity associated with genetically confirmed BBS, loss‐of‐function biallelic pro‐opiomelanocortin (POMC), proprotein convertase subtilisin/kexin type 1 (PCSK1) deficiency, or biallelic leptin receptor (LEPR) deficiency in all patients ≥ 2 years of age [21]. In the United States and Canada, setmelanotide is approved for weight management in all patients ≥ 2 or ≥ 6 years of age with obesity due to BBS or POMC, PCSK1, or LEPR deficiency [22, 23].

In a multicenter, randomized, double‐blind, placebo‐controlled, phase 3 trial, patients with BBS or Alström syndrome ≥ 6 years of age received setmelanotide or placebo in a 14‐week double‐blind period, followed by open‐label treatment with setmelanotide for 52 weeks. In this trial, setmelanotide led to significant reductions in weight and hunger among patients with BBS; 32.3% of patients ≥ 12 years of age reached a ≥ 10% reduction in body weight after 52 weeks of treatment (primary study endpoint) [24]. Treatment was well tolerated, with skin hyperpigmentation being the most commonly reported treatment‐emergent adverse event [24]. These positive effects on body weight persisted for up to 2 years in the long‐term extension study, with no new safety concerns [25]. In the open‐label, multicenter, phase 3 VENTURE trial (NCT04966741), the efficacy of setmelanotide was also established in children 2–5 years of age who had hyperphagia and obesity due to BBS or POMC, PCSK1, or LEPR deficiency. Among the patients with BBS (*n* = 5), four patients (80%) reached a ≥ 0.22‐point reduction in body mass index (BMI) *z*‐score per World Health Organization (WHO) methodology at week 52. All adverse effects were mild or moderate [26].

Real‐world effectiveness of setmelanotide also has been demonstrated. Results from an ongoing prospective monocentric cohort study including 11 patients with BBS in France demonstrated clinically significant weight reductions and improved eating behavior with significant reductions in hunger and food craving [27]. Additionally, an online survey in Germany of 35 patients (including 10 children) with BBS revealed that over 90% of patients reported reduced feelings of insatiable hunger, being satiated after meals, and achieving either a stable body weight or weight loss following treatment with setmelanotide. Moreover, treatment improved mood and behavior [28]. Beyond reductions in feelings of hunger and body weight, setmelanotide also has been associated with improvements in health‐related quality of life [11, 19, 24, 29] and with improvements in estimated glomerular filtration rate and MASLD [30]. The improvements in MASLD were independent of changes in BMI.

Conducting clinical trials in rare diseases is challenging due to the scarcity of patients and ethical considerations regarding placebo use [31]. The blinded placebo‐controlled part of the phase 3 clinical trial for BBS lasted 14 weeks, and longer comparisons with treatment‐naïve patients are not available to further evaluate the clinical benefit of this therapy. An additional consideration with setmelanotide is that hyperpigmentation, a common side effect, makes it challenging to maintain blinding in a double‐blind placebo‐controlled trial.

In this scenario, alternative ways are needed to provide more robust evidence of a treatment effect. In the absence of randomized controlled trials (RCTs) with long‐term follow‐up, indirect treatment comparisons are well accepted to estimate the impact of a therapeutic intervention, especially in rare diseases [32, 33]. Indirect treatment comparisons statistically compare interventions using external control data when head‐to‐head trials are lacking, often by referencing placebo groups from separate studies [34]. When designed carefully, indirect treatment comparisons can fulfill the population, intervention, comparators, and outcomes (PICO) criteria to support decision‐making for both healthcare professionals and regulatory agencies. European recommendations for the performance of indirect treatment comparisons have been published previously [34].

The objective of the current analysis was to further evaluate the clinical benefit of setmelanotide on weight‐related parameters over 52 weeks in patients with BBS and obesity from a phase 3 trial (NCT03746522) compared with a long‐term external control cohort from the international Clinical Registry Investigating BBS (CRIBBS) that received usual care [35].

## 2 Methods

### 2.1 Study Participants

This indirect treatment comparison included patients with BBS from the phase 3 clinical trial (NCT03746522) [24] and patients identified from CRIBBS. All patients from the phase 3 trial who reached ≥ 52 weeks of treatment with setmelanotide were included in this analysis (14 pediatric patients and 15 adults; *n* = 29), including patients who initially received placebo in the 14‐week double‐blind period and then switched over to setmelanotide in the 52‐week open‐label period. Patients were ≥ 6 years of age with a clinical diagnosis of BBS and obesity (defined as BMI > 97th percentile for age and sex for patients 6–15 years of age and ≥ 30 kg/m^2^ for those ≥ 16 years of age). Further eligibility criteria were a ≤ 2% weight loss from intensive diet or exercise regimen (or both) within 2 months prior to enrollment or a ≤ 10% weight loss durably maintained after gastric bypass surgery. Obesity medication was not permitted in the 3 months prior to study enrollment. Moreover, patients were required to have a glomerular filtration rate ≥ 30 mL/min/1.73 m^2^. Clinical and demographic characteristics and the genetic variants identified in the phase 3 trial participants have been published previously [24], with the most common genetic variants being *BBS1* (27.3%) and *BBS10* (25.0%). The trial was performed at 12 sites in the United States, Canada, the United Kingdom, France, and Spain. This study was conducted in accordance with guidelines from the Declaration of Helsinki, Council for International Organizations of Medical Sciences, and International Council for Harmonisation at sites with approval of the Independent Ethics Committee or institutional review board. An Independent Data Monitoring Committee monitored the safety and efficacy data during the study to ensure the highest ethical standards.

The external control group participated in CRIBBS, an international registry and natural history study that records health information and outcomes on a yearly basis in patients with BBS (June 2014 to present [35]). To be eligible for inclusion in the registry, patients must have a clinical diagnosis of four primary features or three primary features plus two secondary features (regardless of genetic findings) or must have a biallelic variant for an established gene associated with BBS [5]. Of the 789 participants in CRIBBS, 728 participants have a clinical diagnosis, and 61 participants have a biallelic variant for an established gene associated with BBS without a clinical diagnosis. All patients enrolled in CRIBBS provided verbal and when applicable written informed consent permitting the collection of their clinical information and its use for research purposes, including clinical analyses and dissemination of results in scientific publications. The study was conducted according to the Declaration of Helsinki and approved by the Marshfield Clinic Research Foundation Institutional Review Board (IRB‐18‐164).

Inclusion criteria from the phase 3 BBS trial were applied to all patients from CRIBBS who were also required to have completed at least one follow‐up questionnaire after the initial phone interview (*n* = 605). Patients from CRIBBS who entered the phase 3 BBS trial were excluded from the comparative cohort. From CRIBBS, 120 patients (51 pediatric and 69 adult) fulfilled the inclusion criteria for this analysis. All but three (pediatric) patients had a clinical diagnosis of BBS at enrollment in the registry.

### 2.2 Treatment Protocol

Patients with BBS from the phase 3 trial received a dose of setmelanotide up to 3.0 mg based on age or placebo during the 14‐week double‐blind period, and they continued open‐label setmelanotide at 3.0 mg for 52 weeks. No participation in diet or exercise interventions or change in drug therapies was allowed during the trial. Further details of the trial protocol have been described extensively elsewhere [24]. In the CRIBBS control cohort, deidentified data collected as part of the registry questionnaires, as well as supplementary information collected from the patients' medical records, were utilized corresponding to the same period as the phase 3 trial (December 10, 2018, to November 25, 2019) to avoid confounding factors, e.g., COVID‐19.

### 2.3 Outcomes

The predefined primary endpoint was the responder rate at 52 weeks. In the pediatric population (patients < 18 years of age at baseline), the response rate was defined as patients who reached a ≥ 0.3‐point reduction in the BMI *z*‐score. The BMI *z*‐score per WHO Child Growth Standard was used to report weight changes in pediatric patients. BMI *z*‐score is a statistical measure to assess BMI in pediatric patients by considering a patient's BMI and comparing it to reference values for the same age and sex. A reduction of ≥ 0.2 points is considered clinically relevant in a pediatric population [36], but a 0.3‐point reduction was used as a stringent responder criterion in this analysis. In the adult population (patients ≥ 18 years of age at baseline), it was defined as patients who had achieved a ≥ 10% reduction in body weight from baseline. Studies have demonstrated that a ≥ 5% weight loss is associated with a clinically meaningful impact on multiple weight‐related diseases [37, 38]. A 10% reduction was used as a stringent responder criterion for adults in this analysis.

Key secondary endpoints were mean change and mean % change in BMI from baseline in the pediatric and adult populations, mean change and mean % change in BMI *z*‐score from baseline in the pediatric population, and mean change and mean % change in body weight from baseline in the adult population. Hunger and quality of life outcomes could not be assessed in this analysis due to incomparable assessments between the phase 3 trial and CRIBBS.

### 2.4 Statistical Analysis

**FIGURE 1:**
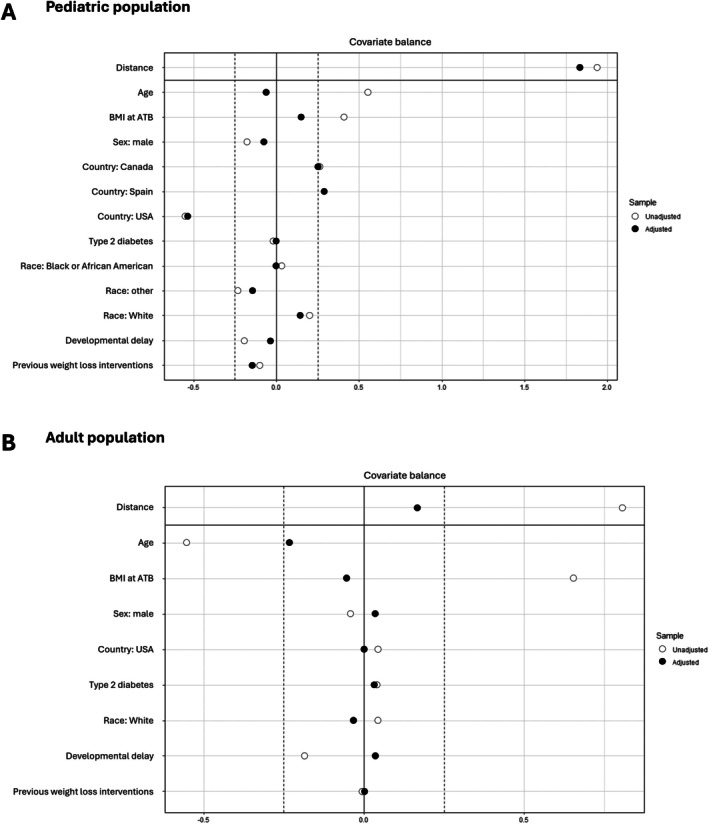
Propensity matching outcomes in the (A) pediatric and (B) adult populations. Standardized mean differences for each covariate before (unadjusted, open circles) and after (adjusted, filled circles) propensity score matching. A covariate was determined as balanced if the value is < 0.25 [40]. ATB, active treatment baseline.

Propensity score matching was applied to reduce the influence of confounding variables and establish two comparable cohorts [39]. Propensity scores were estimated using logistic regression including the covariates of age, BMI, gender, ethnicity, type 2 diabetes, country of residence, cognitive impairment/developmental delay, and previous weight loss interventions (Figure 1). Optimal 2:1 matching without replacement was performed. Figure 1 shows the standardized mean differences for each covariate before (unadjusted, open circles) and after (adjusted, filled circles) propensity score matching. Values of the covariates closer to zero indicate a better balance between the treatment and control groups. A covariate was determined as balanced if the value is < 0.25 [40] The adjusted values demonstrate that matching successfully reduced baseline differences across all listed covariates.

Mean and mean % change from study baseline until week 52 were mapped for all outcomes for both pediatric and adult populations. For patients who received more than 52 weeks of setmelanotide, active treatment baseline (ATB) was considered as the last available measurement before the first dose of setmelanotide. ATB was used to assess a patient's status at the beginning of any setmelanotide treatment, regardless of prior study participation, and differs from study baseline, which may also include a pretreatment or observational period. The controlled treatment effect (CTE) was calculated from ATB until 52 weeks of treatment for all outcomes for both adult and pediatric populations. CTE was calculated with a logistic regression model based on the propensity score matched cohort. CTE estimates the treatment effect after accounting for natural disease progression that would result from no intervention. Unlike placebo‐subtracted values from randomized trials, CTE uses matched control patients from real‐world or historical data to estimate the outcome without treatment [39].

A sensitivity analysis was carried out using inverse probability of treatment weighting (IPTW) to further evaluate the treatment effect of setmelanotide (see online Supporting Information Methods). IPTW is an alternative statistical approach to propensity score matching that involves assigning weights to each subject based on the inverse of the probability of receiving the observed treatment. Unlike propensity score matching, this method uses all available patients rather than creating matched pairs, providing a complementary perspective on treatment effectiveness while accounting for the same baseline characteristics [41]. The IPTW analysis was carried out using the WeightIt package in R. Treatment effects were estimated using a weighted generalized linear model to examine the changes from baseline to follow‐up. Heteroskedasticity‐consistent 3 (HC3) standard errors were used instead of subclass clustering, since IPTW does not generate distinct matched sets. This variance estimator was selected for its robustness in weighted analyses and its conservative properties in finite samples.

All analyses and tabulations were performed using R version 3.1 or higher on a PC platform.

## 3 Results

After matching, a total of 58 patients (28 pediatric and 30 adult patients) from CRIBBS were included in the analysis. Genetic test results were available for 44 patients: 19 (32.8%) pediatric patients and 18 (31.0%) adult patients had a positive genetic test result for a biallelic pathogenic/likely pathogenic variant in BBS‐associated genes (Table 1). The most common genetic variants identified were in *BBS1* (18.9% in pediatric patients, 13.5% in adult patients) and *BBS10* (21.6% in pediatric patients and 21.6% in adult patients).

**TABLE 1:** Biallelic pathogenic/likely pathogenic variants identified in matched patients with BBS from CRIBBS.

Genetic variant, *n* (%)	Adult (*n* = 18)	Pediatric (*n* = 19)	Total (*n* = 37)
BBS1	5 (13.5)	7 (18.9)	12 (32.4)
BBS2	4 (10.8)	2 (5.4)	6 (16.2)
BBS5	0	1 (2.7)	1 (2.7)
BBS9	0	1 (2.7)	1 (2.7)
BBS10	8 (21.6)	8 (21.6)	16 (43.2)
TTC21B	1 (2.7)	0	1 (2.7)

In this analysis, 29 patients (14 pediatric and 15 adult patients) from the phase 3 trial were compared (1:2) with the 58 matched patients from CRIBBS. At baseline, mean BMI *z*‐score in the pediatric population was 3.7 (95% confidence interval [CI]: 2.9, 4.6) in treated patients and 2.5 (95% CI: 2.4, 2.6) in the CRIBBS cohort. Mean BMI at baseline in the adult population was 46.3 kg/m^2^ (95% CI: 43.01, 49.5) in treated patients and 46.58 kg/m^2^ (95% CI: 42.15, 51.0) in the CRIBBS cohort.

**FIGURE 2:**
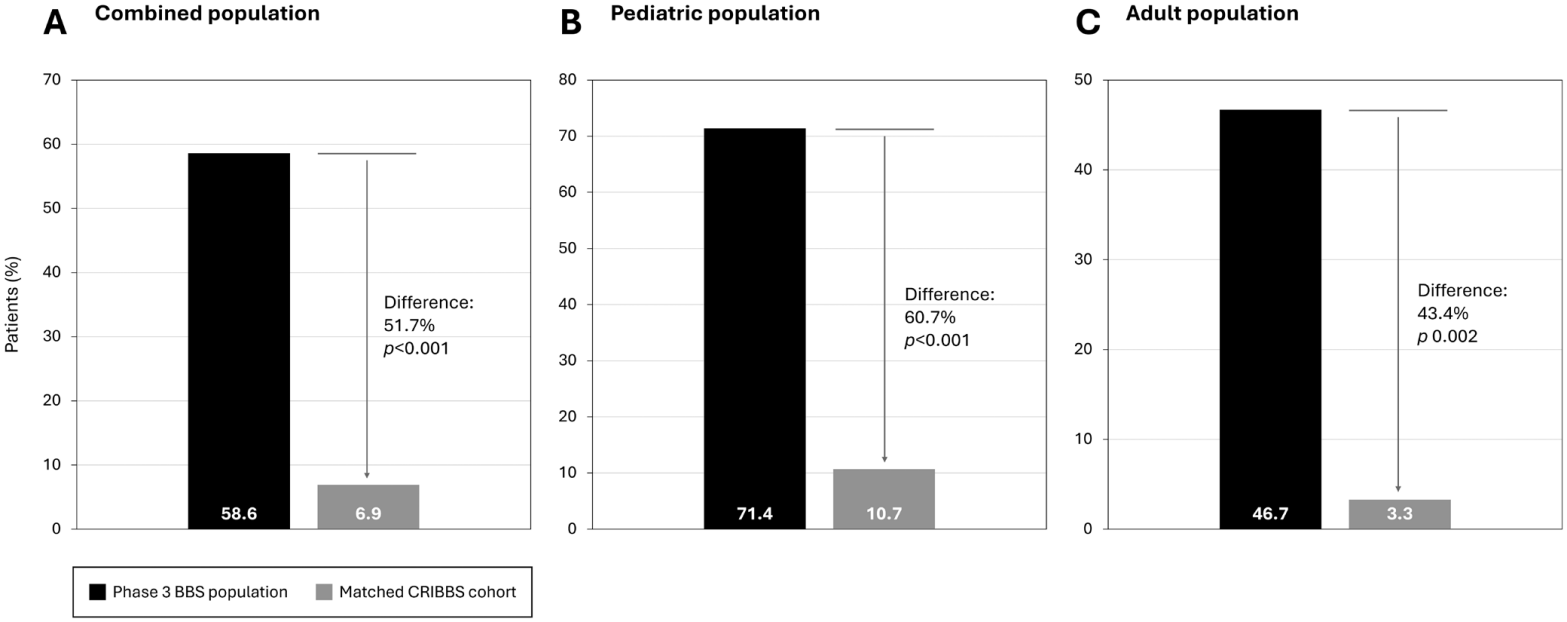
Primary endpoint responder rates in the (A) combined adult and pediatric populations, (B) pediatric population, and (C) adult population. The responder rate was defined as: a ≥ 0.3‐point reduction in BMI *z*‐score in patients < 18 years of age at baseline (pediatric population) or a ≥ 10% reduction from baseline in body weight in patients ≥ 18 years of age at baseline (adult population). BBS, Bardet‐Biedl syndrome; CRIBBS, Clinical Registry Investigating BBS.

Of the combined pediatric and adult treated population, 58.6% (*n* = 17) achieved the primary endpoint compared with 6.9% (*n* = 4) of the CRIBBS cohort, reflecting a 51.7% difference between the two cohorts (*p* < 0.001; Figure 2A). Moreover, 71.4% of treated pediatric patients (*n* = 10) achieved the primary endpoint compared with 10.7% (*n* = 3) of the CRIBBS cohort, reflecting a 60.7% difference between the two cohorts (*p* < 0.001; Figure 2B). In the adult patient population, 46.7% of the treated patients (*n* = 7) achieved this endpoint, compared with 3.3% (*n* = 1) of the CRIBBS cohort, a 43.4% difference between groups (*p* = 0.002; Figure 2C).

Marked benefits of therapy with setmelanotide were also evident for all key secondary endpoints. In the pediatric population (Table 2), mean change in BMI *z*‐score from baseline was −0.8 for treated patients compared with −0.01 in the CRIBBS cohort. The CTE was −0.7 (95% confidence interval [CI]: −1.0, −0.5; *p* < 0.001). Mean % change in BMI *z*‐score from baseline was −24.3% in treated patients compared with −0.3% in the CRIBBS cohort. The CTE was −24.1% (95% CI: −35.7%, −12.4%; *p* < 0.001). Mean change in BMI from baseline was −3.4 kg/m^2^ in treated patients compared with +1.0 kg/m^2^ in the CRIBBS cohort. The CTE was −4.5 (95% CI: −5.7, −3.2; *p* < 0.001). Ultimately, the mean % change in BMI from baseline in the pediatric population was −9.72% for treated patients compared with +3.42% in the CRIBBS cohort. The CTE was −13.1% (95% CI: −17.0%, −9.2%; *p* < 0.001).

**TABLE 2:** BMI outcomes in setmelanotide‐treated patients with BBS compared with the matched CRIBBS cohort—pediatric population.

	BMI, kg/m^2^		BMI *z*‐score	
	Treated BBS cohort	Matched CRIBBS cohort	Treated BBS cohort	Matched CRIBBS cohort
Mean absolute change				
52 weeks	−3.4	1.0	−0.8	−0.01
CTE (95% CI)	4.5 (−5.7, −3.2) *p* < 0.001		−0.7 (−1.0, −0.5) *p* < 0.001	
Mean % change				
52 weeks	−9.7%	3.4%	−24.3%	−0.3%
CTE (95% CI)	−13.1% (−17.0%, −9.2%) *p* < 0.001		−24.1% (−35.7%, −12.4%) *p* < 0.001	

Abbreviation: CTE, controlled treatment effect.

In the adult population (Table 3), the mean change in BMI was −3.3 kg/m^2^ in treated patients compared with +0.4 kg/m^2^ in the CRIBBS cohort. The CTE was −3.7 (95% CI: −5.2, −2.1; *p* < 0.001). The mean % change in BMI was −7.1% in treated patients compared with +0.9% in the CRIBBS cohort. The CTE was −7.9% (95% CI: −11.0%, −4.8%; *p* < 0.001). The mean change in body weight (kg) from baseline in treated patients was −9.4 kg compared with +1.1 kg in the CRIBBS cohort. The CTE was −10.5 (95% CI: −14.9, −6.0; *p* < 0.001). Finally, the mean % change in body weight from baseline in treated patients was −7.6% compared with 0.9% in patients from the CRIBBS cohort. The CTE was −8.5% (95% CI: −11.5%, −5.4%; *p* < 0.001).

**TABLE 3:** Weight outcomes in setmelanotide‐treated patients with BBS compared with the matched CRIBBS cohort—adult population.

	BMI, kg/m^2^		Body weight, kg	
	Treated BBS cohort	Matched CRIBBS control cohort	Treated BBS cohort	Matched CRIBBS control cohort
Mean absolute change				
52 weeks	−3.3	0.4	−9.4	1.1
CTE (95% CI)	−3.7 (−5.2, −2.1); *p* < 0.001		−10.5 (−14.9, −6.0)*; p* < 0.001	
Mean % change				
52 weeks	−7.1%	0.9%	−7.6%	0.9%
CTE (95% CI)	−7.9% (−11.0%, −4.8%); *p* < 0.001		−8.5% (−11.5%, −5.4%); *p* < 0.001	

Abbreviation: CTE, controlled treatment effect.

The sensitivity analysis confirmed the findings of the propensity score matched analysis. Treatment effect estimates and their corresponding confidence intervals were compared and were found to be consistent across two methodological approaches (see Tables S1 and S2). In the pediatric population, IPTW produced similar treatment outcomes to propensity score matching, with BMI *z*‐score changes of −0.8 (95% CI: −1.0, −0.5; *p* < 0.001) versus −0.7 (95% CI: −1.0, −0.5; *p* < 0.001), respectively. Similarly, in the adult population, IPTW demonstrated consistent BMI reductions of −3.7 (95% CI: −5.8, −1.6; *p* < 0.001) compared with −3.7 (95% CI: −5.3, −2.1; *p* < 0.001) with propensity score matching. Additional IPTW sensitivity analyses using trimmed weights and double‐robust estimation supported these findings further, with all methods producing statistically significant treatment effects in the same direction and of a similar magnitude. This strengthens confidence in the validity of observed treatment benefits.

## 4 Discussion

This indirect comparison using a propensity score matched approach demonstrates the significant efficacy of setmelanotide in reducing obesity‐related parameters in patients with BBS, offering compelling additional support for the therapeutic benefits of this treatment. The propensity score matched approach with carefully selected covariates minimized confounding and strengthened internal validity; in addition, the validity of these results was corroborated in the sensitivity analysis using IPTW. These results contribute to understanding the effects of setmelanotide over 52 weeks and reinforce its clinically relevant benefits compared with usual care.

Although the efficacy and safety of setmelanotide have been demonstrated previously, the blinded placebo‐controlled part of the phase 3 trial lasted only 14 weeks. CRIBBS provided the opportunity to analyze a large cohort of patients with BBS, allowing an indirect comparison of setmelanotide versus usual care over 52 weeks. At 52 weeks, patients treated with setmelanotide exhibited significant and clinically meaningful improvements in weight‐related primary and secondary endpoints compared with propensity score matched patients from CRIBBS. The primary endpoint was met by a significantly larger proportion of patients treated with setmelanotide compared with control patients. Secondary endpoint analyses further substantiated the therapeutic benefit of setmelanotide; reductions in weight‐related outcomes were consistently greater in the patients with BBS treated with setmelanotide compared with control patients from CRIBBS. Across all endpoints, the CTE remained robust and statistically significant.

Participation in registries has been shown to enhance health care engagement by fostering better communication, transparency, and empowerment in decision‐making [40]. This benefit is reflected in an analysis from CRIBBS, which included 331 pediatric patients with BBS [12]. At baseline, 81% of these patients had obesity, and 22.7% reported using weight loss diets or medications. Despite the availability of interventions, including weight‐reducing medications, only a small proportion of patients from CRIBBS achieved clinically meaningful weight loss compared with setmelanotide‐treated patients in this indirect comparison. This comparison highlights the significant challenge that patients with BBS face without therapies specifically targeting hyperphagia and concomitant obesity.

An important distinction between the source datasets is that patients in the phase 3 BBS trial were not permitted to initiate or modify weight loss pharmacotherapy or to optimize lifestyle interventions during the blinded or open‐label treatment periods. In contrast, participants in CRIBBS could make such changes as part of usual care. Although detailed information on how frequently, or to what extent, these interventions occurred in CRIBBS is not available, this difference in study design has the potential to influence outcomes in favor of the control group. The fact that greater improvements were still observed in the setmelanotide group should be interpreted in this context.

Using a real‐world comparator is especially valuable in rare diseases where data from long‐term randomized trials is difficult to obtain due to limited populations and ethical concerns. This approach aligns with recent Coordination Group on Health Technology Assessment and European Medicines Agency recommendations for leveraging indirect treatment comparisons in rare conditions [34]. Employing a well‐characterized, external natural history cohort (CRIBBS) enhances the generalizability of findings, supporting the effectiveness of setmelanotide beyond the confines of a controlled trial. An additional consideration with setmelanotide is that hyperpigmentation, a common side effect, makes it challenging to maintain blinding in a double‐blind placebo‐controlled trial.

While the treatment effect of setmelanotide on hunger, quality of life, and metabolic outcomes would offer a more comprehensive picture of its long‐term benefits, there were no comparable assessments possible between the phase 3 trial and CRIBBS.

## 5 Conclusion

This indirect treatment comparison using a propensity score matched cohort from CRIBBS provides strong evidence that setmelanotide offers clinically and statistically significant weight‐related benefits over 52 weeks in both pediatric and adult patients with BBS, reinforcing its long‐term therapeutic value compared with usual care. Early intervention in patients with BBS and hyperphagia and obesity should be endorsed to help improve weight‐related outcomes and reduce the risk of obesity‐related complications and associated impacts on quality of life.

## Funding

This research was funded by Rhythm Pharmaceuticals, Inc. The Clinical Registry Investigating Bardet Biedl Syndrome (CRIBBS) is funded by a grant from the Bardet Biedl Syndrome Foundation.

## Conflicts of Interest

Jesús Argente has received compensation for speaking engagements and institutional study funding from Rhythm Pharmaceuticals, Inc., as a clinical investigator and participated in the BBS advisory board for Rhythm Pharmaceuticals. Andrea M. Haqq is an advisory board member for Novo Nordisk, Rhythm Pharmaceuticals, and The Foundation for Prader‐Willi Research USA; a grant recipient for Weston Family Microbiome Initiative and Canadian Institutes of Health Research; and Primary Investigator on clinical trials with Aardvark Therapeutics, Acadia Pharmaceuticals, Eli Lilly, Novo Nordisk, and Rhythm Pharmaceuticals. Jeremy Pomeroy has received compensation for speaking engagements and institutional study funding from Rhythm Pharmaceuticals as a co‐investigator and participated in an advisory board for Rhythm Pharmaceuticals. Jan Luca Schorfheide and Urs Wiedemann are employees of stradoo GmbH, contracted by Rhythm Pharmaceuticals to perform the analyses. Nicolas Touchot and Caroline Huber are employees at Rhythm Pharmaceuticals and have received salary and stock options for their employment.

## Supporting information


**TABLE S1:** IPTW analysis outcomes in the pediatric population.
**TABLE S2:** IPTW analysis outcomes in the adult population.

## Data Availability

The data that support the findings of this study are available from the corresponding author upon reasonable request.

## Appendix 1

Contributors included in the Phase 3 BBS Trial Investigators include: Dr. Wendy Chung, Boston Children's Hospital, Boston, Massachusetts, USA; Professor Karine Clément, Sorbonne Université and Pitié‐Salpêtrière Hospital, Paris, France; Professor Hélène Dollfus, INSERM‐Université de Strasbourg and Hôpitaux Universitaires de Strasbourg, Strasbourg, France; Dr. Elizabeth Forsythe, National Bardet‐Biedl Syndrome Clinic, Guys and St Thomas' Hospital, National Bardet‐Biedl Syndrome Clinic, Great Ormond Street Hospital, London, UK; Dr. Robert M. Haws, Marshfield Clinic Research Institute, Marshfield, Wisconsin, USA; Dr. Gabriel Á. Martos‐Moreno, Hospital Infantil Universitario Niño Jesús, Madrid, Spain; Christine Poitou, Sorbonne Université and Pitié‐Salpêtrière Hospital, Paris, France; and Dr. Jack A. Yanovski, Eunice Kennedy Shriver National Institute of Child Health and Human Development, National Institutes of Health, Bethesda, Maryland, USA. Dr. Jack A. Yanovski, contributor to this publication, is supported by the Intramural Research Program of the National Institutes of Health (NIH). The contributions of the NIH author are considered Works of the United States Government. The findings and conclusions presented in this paper are those of the authors and do not necessarily reflect the views of the NIH or the U.S. Department of Health and Human Services.
