# Supplementary material for: Setmelanotide in Bardet‐Biedl Syndrome: A 52‐Week Comparison of Phase 3 Trial Participants With a Matched Registry Cohort
Source: Obesity (Silver Spring). 2026 Feb 17;34(3):579–87. doi: 10.1002/oby.70125 (PMC12933228; doi:10.1002/oby.70125)
Supplement: Supplementary file 1 — TABLE S1: IPTW analysis outcomes in the pediatric population. TABLE S2: IPTW analysis outcomes in the adult population. [file OBY-34-579-s001.docx]

## Supporting Information

**Supplemental methods**

Inverse probability of treatment weighting (IPTW) was conducted as a sensitivity analysis to complement the primary propensity score (PS) matching approach used to create the external control arm. IPTW assigns weights to each subject based on the inverse of their probability of receiving their observed treatment [41]. This creates a pseudo-population in which measured confounders are balanced between groups. Specifically, treated patients receive a weight of 1/PS, and control subjects receive a weight of 1/(1−PS). Unlike matching, which may exclude unmatched observations, IPTW retains all subjects while achieving covariate balance through weighting. This preserves both statistical power and the representativeness of the original sample. This methodological triangulation strengthens causal inference by providing an alternative approach to addressing selection bias that relies on different statistical assumptions to those of PS matching [41]. The analysis included the same pre-treatment covariates as the primary analysis: age, baseline BMI, sex, country, type 2 diabetes status, race, developmental delay, and previous weight-loss interventions. Treatment effects were estimated using weighted generalized linear models with HC3 standard errors, which provide robust variance estimation in finite samples.

To further enhance the robustness of our findings, two additional sensitivity analyses were performed: weight trimming at the 99th percentile to mitigate the influence of outliers while maintaining balance, and double-robust estimation combining IPTW with covariate adjustment in the outcome model to protect against misspecification of the treatment assignment or outcome model. Consistent findings across PS matching, standard IPTW, trimmed IPTW, and double-robust approaches provide strong evidence for the validity of treatment effect estimates in both pediatric and adult populations.

**TABLE S1.** IPTW analysis outcomes in the pediatric population.

| Outcome | Method | Estimate | CI | *p* value |
| --- | --- | --- | --- | --- |
| BMI z-score change | Propensity score matching | −0.74 | [−0.99, −0.5] | <0.001 |
|  | IPTW | −0.75 | [−1.01, −0.49] | <0.001 |
|  | IPTW - trimmed weights | −0.75 | [−1.01, −0.49] | <0.001 |
|  | IPTW - double-robust | −0.91 | [−1.07, −0.75] | <0.001 |
| BMI z-score % change | Propensity score matching | −0.24 | [−0.36, −0.12] | <0.001 |
|  | IPTW | −0.24 | [−0.37, −0.12] | <0.001 |
|  | IPTW - trimmed weights | −0.24 | [−0.37, −0.12] | <0.001 |
|  | IPTW - double-robust | −0.28 | [−0.36, −0.19] | <0.001 |
| BMI change | Propensity score matching | −4.47 | [−5.71, −3.22] | <0.001 |
|  | IPTW | −5.08 | [−7.03, −3.13] | <0.001 |
|  | IPTW - trimmed weights | −5.08 | [−7.03, −3.123 | <0.001 |
|  | IPTW - double-robust | −6.19 | [−7.91, −4.46] | <0.001 |
| BMI % change | Propensity score matching | −0.13 | [−0.17, −0.09] | <0.001 |
|  | IPTW | −0.14 | [−0.20, −0.09] | <0.001 |
|  | IPTW - trimmed weights | −0.14 | [−0.20, −0.09] | <0.001 |
|  | IPTW - double-robust | −0.18 | [−0.22, −0.13] | <0.001 |

BMI, body mass index; CI, confidence interval; IPTW, inverse probability of treatment weighting.

**\**

**TABLE S2.** IPTW analysis outcomes in the adult population.

| Outcome | Method | Estimate | CI | *p* value |
| --- | --- | --- | --- | --- |
| BMI change | Propensity score matching | −3.65 | [−5.25, −2.06] | <0.001 |
|  | IPTW | −3.71 | [−5.80, −1.61] | <0.001 |
|  | IPTW - trimmed weights | −3.67 | [−5.77, −1.58] | <0.001 |
|  | IPTW - double-robust | −3.66 | [−5.94, −1.37] | <0.001 |
| BMI % change | Propensity score matching | −0.08 | [−0.11, −0.05] | <0.001 |
|  | IPTW | −0.08 | [−0.12, −0.04] | <0.001 |
|  | IPTW - trimmed weights | −0.08 | [−0.12, −0.04] | <0.001 |
|  | IPTW - double-robust | −0.08 | [−0.13, −0.03] | <0.001 |
| Weight change | Propensity score matching | −10.47 | [−14.93, −6.01] | <0.001 |
|  | IPTW | −10.47 | [−16.14, −4.81] | <0.001 |
|  | IPTW - trimmed weights | −10.38 | [−16.04, −4.71] | <0.001 |
|  | IPTW - double-robust | −10.39 | [−16.62, −4.15] | <0.001 |
| Weight % change | Propensity score matching | −0.09 | [−0.12, −0.05] | <0.001 |
|  | IPTW | −0.08 | [−0.13, −0.04] | <0.001 |
|  | IPTW - trimmed weights | −0.08 | [−0.13, −0.04] | <0.001 |
|  | IPTW - double-robust | −0.08 | [−0.13, −0.04] | <0.001 |

BMI, body mass index; CI, confidence interval; IPTW, inverse probability of treatment weighting.
